# Weekly temperature data are sufficient to estimate exposure-response relationships: a boon for health adaptation in low-resource settings

**DOI:** 10.1016/j.lanepe.2023.100811

**Published:** 2023-11-29

**Authors:** Kristie L. Ebi

**Affiliations:** University of Washington, USA

In this issue of *The Lancet Regional Health—Europe*, Ballester et al. analyzed temperature and mortality records for 147 regions in 16 European countries to determine whether weekly, biweekly, or monthly aggregation of mortality data could provide robust estimates of the short-term effects of exposure to high and low ambient temperatures.^1^ The standard has been to use daily data,^2^ but such data are generally unavailable in low-resource settings. This means there are very few quantifications for these countries and regions, leading to underestimates of their risks and to the incorrect perspective that high income countries are more affected. Providing such quantifications is critical to prepare for and manage exposure to temperature extremes, including through implementing early warning and response systems, and for research and policymaking.

The analyses by Ballester et al. bring the welcome news that weekly mortality and temperature data reasonably estimated exposure-response relationships, when compared with daily data.^1^ Bi-weekly data systematically and significantly underestimated heat-related mortality (46%) and monthly data were too course to estimate the short-term impact of extreme temperature on mortality. Further, because linear trends were generally preserved, weekly data can be used to construct indicators to track heat-related mortality, both to understand impacts over time and to estimate the effectiveness of adaptation inventions. Understanding which interventions are most effective and efficient can better focus adaptation investments.

Adaptation is increasingly important in a changing climate. The first three quarters of 2023 were exceptionally warm, putting the year on track to be the warmest year since records began in the mid-1800s.^3^ So far in 2023, extreme heatwaves were experienced in the UK, large parts of Europe, southern US and Mexico, Central America, South America, Caribbean, Korea, Japan, and China. These caused not just preventable heat-related deaths but affected birth outcomes and reduced worker productivity.^4^ The frequency, intensity, and duration of heatwaves are projected to increase with additional climate change, putting more people at risk.^5^

Because nearly all heat-related deaths are preventable, heatwave early warning and response systems are key for protecting and promoting health.^6^ These systems are based on location-specific thresholds for action. In most locations, access to health data from national registries is more limited than for temperature data, particularly in low- and middle-income countries. Weekly health data are more widely available than daily data, including through some open access databases.

Complexities in establishing thresholds for action include that the temperature at which mortality is at its lowest varies across locations, with linear relationships between temperature and daily mortality above/below this temperature. These linear relationships make choosing temperature thresholds challenging. As with other continuous variables, such as blood pressure, thresholds need to balance selecting too low a temperature that results in warning fatigue and selecting too high a temperature that then fails to prevent avoidable deaths.^7^ Detailed local analyses are needed to inform this decision; the lack of daily data is a barrier to action, condemning individuals to preventable deaths.

Knowing that weekly data are sufficient can support the World Meteorological Organization Early Warnings for All initiative.^8^ With an investment of over USD 3 billion, the initiative identified ambitious goals over the next four years to ensure that all people on Earth are protected from hazardous weather and climate events through furthering development of early warning systems. One focus will be heatwave early warning and response systems to improve health and well-being.^9^

One caveat is the degree of underestimation when using weekly data. The weekly model underestimated heat-related mortality by over 20%, but only underestimated mortality from extreme temperature events by under 5%.^1^ The absolute differences between the daily and weekly data models were relatively constant across the year. Additional research in other regions would increase the precision of that estimate so that a constant can be applied to increase the accuracy of estimated mortality in locations with only weekly data. Another caveat is that the analyses did not include consideration of air pollution; a knowledge gap that can be filled with further research.

Taking advantage of the knowledge that weekly data can provide robust estimates of the risks of exposure to extreme temperatures requires building the capacity of health information systems for reliable and accurate data collection. This is further incentive for investments in integrated surveillance systems in collaboration with national hydrometeorological services. Building this capacity can move research and implementation forward to save the health, well-being, and productivity of billions of people, even as temperatures continue to rise.

## Declaration of interests

None.
